# Correction: Chemical degradation as an enabling pathway to polymersome functionalization

**DOI:** 10.1039/d5ra90108a

**Published:** 2025-10-01

**Authors:** Chenyu Lin, Kumar Siddharth, Juan Pérez-Mercader

**Affiliations:** a Department of Earth and Planetary Sciences, Harvard Origins of Life Initiative, Harvard University Cambridge MA 02138-1204 USA chenyu_lin@fas.harvard.edu jperezmercader@fas.harvard.edu; b Santa Fe Institute Santa Fe NM 87501 USA

## Abstract

Correction for ‘Chemical degradation as an enabling pathway to polymersome functionalization’ by Chenyu Lin *et al.*, *RSC Adv.*, 2025, **15**, 4693–4700, https://doi.org/10.1039/D4RA08536A.

The authors regret that within the first two lines of the abstract, some words have been used incorrectly, and this has altered the meaning.

Correct opening lines of abstract:

‘Self-reproduction and the ability to functionalize are basic features of natural living systems. Understanding the chemical roots of functionalization is fundamental for the generation of new materials in the laboratory and chemistry-based natural-life-mimicking artificial or synthetic living systems.’

The Royal Society of Chemistry apologises for these errors and any consequent inconvenience to authors and readers.

